# Recent Advances in Food Safety: Nanostructure-Sensitized Surface-Enhanced Raman Sensing

**DOI:** 10.3390/foods14071115

**Published:** 2025-03-24

**Authors:** Zeyan Liu, Renqing Yang, Haili Chen, Xinai Zhang

**Affiliations:** School of Food and Biological Engineering, Jiangsu University, Zhenjiang 212013, China; 2212318015@stmail.ujs.edu.cn (Z.L.); 2212318006@stmail.ujs.edu.cn (R.Y.); 2212218017@stmail.ujs.edu.cn (H.C.)

**Keywords:** surface-enhanced Raman scatter, nanostructures, sensing platform, chemical mechanism, food safety, hazardous substances

## Abstract

Food safety is directly related to human health and has attracted intense attention all over the world. Surface-enhanced Raman scattering (SERS), as a rapid and selective technique, has been widely applied in monitoring food safety. SERS substrates, as an essential factor for sensing design, greatly influence the analytical performance. Currently, nanostructure-based SERS substrates have garnered significant interest due to their excellent merits in improving the sensitivity, specificity, and stability, holding great potential for the rapid and accurate sensing of food contaminants in complex matrices. This review summarizes the fundamentals of Raman spectroscopy and the used nanostructures for designing the SERS platform, including precious metal nanoparticles, metal–organic frameworks, polymers, and semiconductors. Moreover, it introduces the mechanisms and applications of nanostructures for enhancing SERS signals for monitoring hazardous substances, such as foodborne bacteria, pesticide and veterinary drug residues, food additives, illegal adulterants, and packaging material contamination. Finally, with the continuous progress of nanostructure technology and the continuous improvement of SERS technology, its application prospect in food safety testing will be broader.

## 1. Introduction

Food safety is a critical issue in the global public health domain, directly impacting consumer health [1,2,3]. With the increasing complexity of modern food production and supply chains, the contamination of harmful substances in food has become more pronounced [4,5]. The harmful substances are mentioned as microorganisms [6,7,8,9,10], chemical substances [11,12,13,14,15], and physical contaminants [16,17,18,19] that may be introduced during food harvest, processing, storage, and sale [20,21,22,23,24,25]. As mentioned, microbiological contamination, such as bacteria [3,26,27,28,29,30], viruses [31,32,33,34], and fungi [35,36,37,38], can lead to outbreaks of foodborne diseases, posing significant challenges to public health safety [23,39,40]. Chemical contamination, including residues of veterinary drugs [39,41,42] and pesticides [43,44,45,46], heavy metals [47,48,49,50,51,52], food additives [53,54,55,56], and illegal adulterants [57,58,59], is also an issue that cannot be overlooked in food safety. A long-term intake of these chemical substances may have chronic effects on human health, including carcinogenesis, neurological damage, and other health issues [60,61]. Physical contamination, such as glass, metal, rocks, fish bones [62], and packaging contamination [17,18], although not usually causing acute health risks, can cause physical injury and discomfort to consumers.

To protect consumers from the potential hazards, governments and international organizations have established food safety standards [63,64,65,66]. Although the accuracy of traditional methods in food safety assessments is high, they often have some limitations, such as high cost, long duration, laborious operation, and an inability to achieve on-site analysis [67,68,69,70]. Therefore, the development of rapid and sensitive technologies is crucial for improving the efficiency of food safety management. Fortunately, surface-enhanced Raman scattering (SERS) is a fast and sensitive fingerprint vibrational spectroscopy with promising potential for the non-destructive and single-molecule detection of components in food. The physicochemical properties of the SERS substrate determine the sensing performance and reliability of the detection method [71,72,73,74]. Although single precious metal SERS substrates often produce higher reinforcement factors through strong electromagnetic enhancement, they still have obvious shortcomings, such as high cost, easy aggregation, and poor affinity with biomolecules and organic pollutants. These defects in mono-precious metal SERS substrates often lead to an unsatisfactory or unreliable SERS performance in complex situations, such as food inspection, hindering the expansion of SERS inspection technology to the food industry. In order to get rid of the dilemma of SERS being “locked” by precious metals, the researchers proposed nanohybridization solutions to develop novel nanostructure-sensitized SERS substrates that combine plasmonic precious metals with multifunctional functional materials, such as magnetic nanoparticles, semiconductors, two-dimensional nanomaterials, metal–organic backbone materials (MOFs), and biopolymers. Compared with single precious metal SERS substrates, these hybrid materials not only synergistically enhance SERS activity through the plasma coupling effect or CM effect, but also endow the substrate with useful functions, such as magnetism, adsorption, molecular sieve, target affinity, etc. [21,75,76,77,78,79]. In recent years, the design of many versatile hybrid SERS substrates has also been innovated to advance sensor development. Therefore, it is of broad and timely significance to review the prospects of nanohybridization-enhanced SERS technology in food safety assurance. However, a comprehensive understanding of the design, fabrication, and functionality of nano-nanostructure-sensitized SERS platforms remains missing in the reported literature.

To fill this gap, this review provides an overview of the progress in the nanostructure-sensitized SERS platform, with a focus on the function-directed design of nanostructure-sensitized SERS substrates for applications that detect the microbiological [80,81,82], chemical [45,83,84,85,86], and physical contamination [87] of food. First, this paper introduces the principle and enhancement mechanism of SERS and points out the advantages of SERS technology in food safety testing. Then, the materials, types, and enhancement mechanisms of nanostructure-sensitized SERS substrates were summarized. Next, the representative applications of the nanostructure-sensitized SERS platform in food safety testing are discussed in detail. It is hoped that this paper can provide valuable ideas for the recent research and development of high-performance SERS platforms in food and other fields.

## 2. Analytical Mechanism of SERS

### 2.1. Basic Theory of SERS

**Figure 1:**
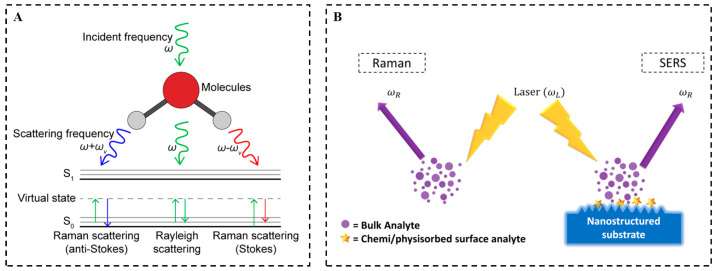
(**A**) Schematic diagram of the Raman scattering effect [91]. (**B**) Schematic comparison of the Raman and SERS phenomena [88].

As is known, when a beam of light strikes an uneven substrate, the light can be reflected, scattered, or absorbed. During the mentioned process, the scattered part undergoes a change in frequency, it is named as Raman scattering [88], or the frequency remains unchanged: this is Rayleigh scattering. Photon scattering of Rayleigh scattering accounts for about 99.9%, Raman scattering about 0.1%. From a microscopic perspective, when an excited molecule absorbs the energy of a photon, it can transition to a virtual state, and then return to the ground state, resulting in Rayleigh scattering. Nevertheless, when the molecule reaches another energy level, it is called Raman scattering, in which it can be classified as anti-Stokes scattering (the molecule absorbs energy) and Stokes scattering (the molecule loses energy) (Figure 1A) [89]. The energy difference between two photons is presented as Equation (1):

(1) $$∆E=h∆v=h\left(v-v^{\prime }\right),$$

where ∆v represents the frequency shift in the Raman scattering spectrum.

Unlike typical Raman spectra, the intensity of SERS spectra is dependent on the interaction of rough metal surfaces (Ag or Au nanoparticles) with the target molecule (Figure 1B) [90]. Specifically, SERS has the following characteristics: (1) high sensitivity, with an intensity that is 7 to 14 orders of magnitude greater than that of spontaneous Raman scattering; and (2) excellent selectivity, with the selective amplification of certain normal vibrations associated with a given electronic absorption band. With these merits, SERS offers an effective technique for studying the vibrational and electronic structure of biomolecules, which can be applied in the fields of life sciences and food.

**Figure 2:**
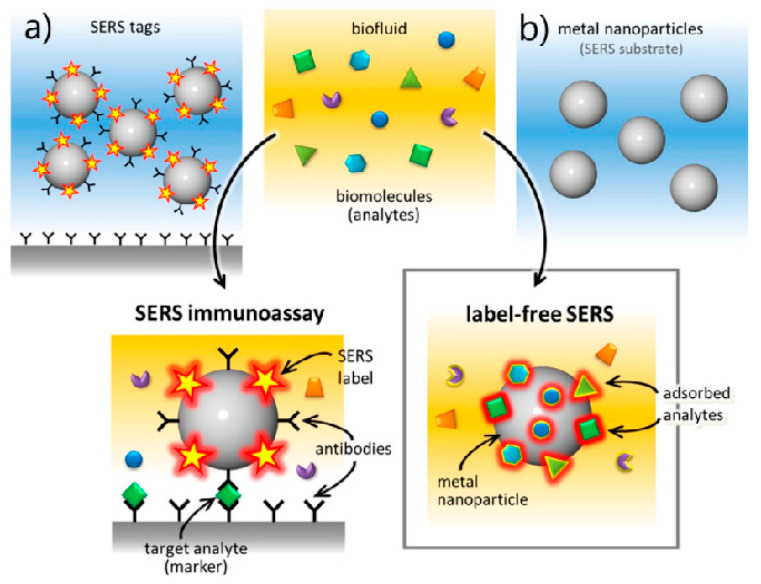
(**a**) Indirect protocol. SERS tag with antibodies for selectively binding to the analyte and Raman reporter for the signal output. (**b**) Direct protocol. The analyte is adsorbed on the nanostructures and detected through its own Raman spectra [96].

According to whether the molecule (e.g., 4-Mercaptobenzoic acid (4-MBA), 4-aminothiolphenol (4-ATP)) is labeled with Raman or not, SERS patterns are categorized into two main strategies: label-free (direct) and label-based (indirect) SERS (Figure 2). As for the former, label-free SERS can trace the intrinsic fingerprint of an analyte through its direct interaction with the sensing substrate, possessing the merits of simplicity, rapidity, cost-effectiveness, and a lack of interference from other components. As for the latter, label-based SERS exploits signal tags that consist of specific Raman reporter molecules for capturing analytes. This approach offers the benefits of multiplex analysis, enhanced sensitivity, and improved repeatability over the label-free pattern [92,93,94,95].

SERS enhancement is accomplished through two mechanisms: electromagnetic (EM) and chemical enhancement (CM) [97,98]. The main point in the EM mechanism is to couple incident light with plasmonic nanostructure LSPRs such that the secondary electric field efficiently concentrates the EM field. When molecules are in close vicinity to plasmonic nanostructures, the amplified EM field enhances the SERS intensity. The EM mechanism is considered to be the predominant contributor to SERS signal amplification, and the *EF* can be as high as 10^10^–10^11^. When molecules bind directly to the plasmonic surface to form a charge–transfer structure, CM is associated with the intrinsic chemical composition of the analyte, with the *EF* usually in the order of 10^1^ to 10^2^ [99]. The collective effects of the EM and CM on SERS signal intensity (*I_SERS_*) can be articulated as shown in Equation (2) [100,101].

(2) $$I_{SERS}{\propto EF}_{EM}\times {EF}_{CM}\times N\times I_{0},$$

where *I*_0_ is the incident light intensity, *I_SERS_* is the strength of the SERS signal, and *N* is the number of Raman probe molecules irradiated on sensing substrate. The SERS performance of the substrate can be evaluated by *EF*, where the *EF_EM_* caused by the EM co ponent can be calculated by the following Equation (3):

(3) $${EF}_{EM}=\frac{{{|E}_{LOC}(\omega_{L})|}^{2}{{|E}_{LOC}(\omega_{R})|}^{2}}{|{E_{INC}|}^{4}},$$

where *E_LOC_* is the intensity of the local electric field; *E_INC_* is the intensity of the incident light field; *ω_L_* is the frequency of incident light; and *ω_R_* is the frequency of Raman scattered light. When the *ω_L_* is very close to the *ω_R_*, the above equation can be approximated as Equation (4).

(4) $${EF}_{EM}=\frac{{{|E}_{LOC}(\omega_{L})|}^{4}}{|{E_{INC}|}^{4}},$$

Here, the *EF* is roughly related to the fourth power of the ratio of the *E_LOC_* to the *E_INC_*.

The *EF* induced by CM enhancement has the following relationship with the charge transfer (CT) mechanism (Equation (5)):

(5) $${EF}_{CM}\sim ({\frac{1}{\Gamma })}^{4},$$

where *Γ* is the half-height width of the absorption peak of the electron transition obtained from the molecular absorption spectrum, and *EF_CM_* is the enhancement factor caused by CM enhancement.

As seen from the above formula, *EF_EM_*, *EF_CM_*, *N*, and *I*_0_ are the key factors regarding SERS signal strength. When calculating the total *EF*, *EF_EM_* and *EF_CM_* are usually not separated, and the *EF* value can be calculated according to the following equation:

(6) $$EF=\frac{I_{SERS}/N_{SERS}}{I_{NOR}/N_{NOR}},$$

(7) $$I_{SERS}{=N}_{A}S_{LAS}VC_{SERS}/S_{CON},$$

(8) $$I_{NOR}{=N}_{A}S_{LAS}VC_{NOR}/S_{CON}.$$

where *N_NOR_* is the molecule number in the laser irradiation volume, *N_SERS_* is the total number of molecules adsorbed on the sensing platform, *N_A_* is the Avogadro’s constant, and *S_LAS_* represents the laser spot area. *V* is the volume of the solution, *S_CON_* is the contact area of the molecules, *I_SERS_* indicates the Raman signal strength of the molecule to be tested based on the enhanced substrate, and *I_NOR_* is the Raman signal strength obtained from the normal substrate. *C_SERS_* is the molecular concentration based on the SERS substrate. *C_NOR_* is based on the molecular concentration of the normal substrate [100,101].

### 2.2. Raman Signal Acquisition

Raman microscopy [92], which integrates Raman spectroscopy with an optical microscope, enables the acquisition of Raman scattering through an objective lens. Meanwhile, confocal Raman microscopy directs a point light source through a pinhole and beam splitter, focusing it to the diffraction-limited spot on the specimen via and objective lens. The scattered or emitted light is collected, collimated, and passed through a detector pinhole before reaching a spectrometer. The detector pinhole serves as the depth selector, rejecting out-of-focus light and selectively gathering signals from the focal plane. Especially, confocal microscopes have emerged as valuable analytical tools, offering a superior depth resolution and enhanced image contrast through the suppression of stray light.

Raman imaging can exhibit the chemical composition by integrating spatial (x, y dimensions) and spectral (wavelength dimension) information from Raman spectroscopy. There are two primary Raman imaging methodologies: scanning and wide-field imaging. Scanning imaging is usually assisted by a confocal microscope, with both point scanning and line scanning. Point scans sequentially collect Raman spectra for each spatial location. The sample is moved across a high-precision stage, controlling both the lateral and axial positions. It offers a high spectral resolution and full spectral capture, but is time-intensive and susceptible to laser-induced sample damage [92]. Line scans use laser lines to broaden the spatial range of each scan, and each measurement collects a line of spatial and spectral data. The sample is automated along a stage axis perpendicular to the laser line. Despite the reduced Raman signal intensity, line scanning maintains a high spectral resolution and is more time-efficient than point scanning. Regarding wide-field Raman imaging, the entire sample area is simultaneously illuminated, enabling the acquisition of spatial data in a single, non-contact scan. Among the developed wide-field imaging, area scanning involves the selection of a specific Raman scattering spectral slice for analysis [92]. While this approach is fast, it is limited by the complexity of spectral data discrimination.

### 2.3. SERS Superiority

The primary constraint of traditional Raman spectroscopy is the inherently weak signal, with only about 1 in 10^6^ to 10^8^ photons being inelastically scattered. This limitation can impede the detection of molecules at low concentrations, thereby restricting its utility in food industry applications [102]. SERS overcomes the shortcomings by employing a metal-based sensing platform, greatly amplifying the Raman signal of even a single molecule by several orders of magnitude, typically from 10^7^ to 10^14^. To be precise, when studying single-molecule-enhanced Raman signals, the theoretical electromagnetic field enhancement under optimal conditions is only 11–12 orders of magnitude. However, the Raman enhancement that was observed experimentally can be up to 14 orders of magnitude, which is still several orders of magnitude of the theoretical electromagnetic field enhancement. The assay principle involves the interaction between rough metal surfaces (e.g., Au, Ag, Cu) and target molecules (e.g., fungus, *Escherichia coli* (*E. coli*)), in which the inelastic light scattering of the target molecules is enhanced [1,97]. With the continuous development of nanotechnology, various organic/inorganic metal, transition metal, and non-metal nanomaterials have been found to exhibit unique physicochemical properties, which have the potential to reconstruct plasmonic nanostructures or exert intrinsic SERS activity. The researchers prefer to hybridize two or more functional nanomaterials with different advantages [103,104,105], such as different precious metals, magnetic nanomaterials, MOFs, nanozymes, etc., to fabricate multifunctional nanostructured SERS substrates compared to single precious metal SERS substrates, which combine the advantages of a single material and exhibit multiple functional properties [106,107]. For example, gold–silver hybrid materials combine the chemical stability and biocompatibility of Au with the strong plasmon resonance effect of Ag. Magnetic hybrid materials display fast magnetic separation and magnetron hot spot reforming [108]. Two-dimensional hybrid materials have high stability and a large specific surface area, which not only enrich targets, but also act as carriers to fix plasmonic nanoparticles and generate hot spots in longitudinal nanogaps [109,110]. In addition, they include nano-coated hybrid materials with high stability and biocompatibility. Green, highly flexible, and easily modifiable biopolymer hybrid materials have a low cost, high stability, and modifiable non-precious metal hybrid materials [111,112,113]. Functional materials can play an important role in hot spot construction, analyte capture, and chemical enhancement through structural features and unique optoelectronic properties. They provide solutions to the problems of weak multi-target analysis, weak selectivity, poor stability, difficult recovery, and low reproducibility in traditional single-metal precious metal SERS substrates.

Moreover, samples can be detected with or without simple pretreatment with SERS. For example, only organic reagents are added to the outside of the peel for extraction, and colloidal SERS substrates are added for contact adsorption. This allows the pesticide to be extracted from the peel matrix, allowing the direct detection of the pesticide of interest without any optimized handling. In addition, the advent of portable Raman spectroscopy provides a platform for the application of SERS technology in this field, and there are increasing reports that it is feasible to use portable Raman spectroscopy for the rapid analysis of trace contaminants [114]. At the same time, portable SERS substrates have been developed, such as paper substrates, adhesive tapes, and composite films. They can be applied to SERS analysis with a simple wipe or dipping, which greatly simplifies the process and reduces the analysis time [115]. These procedures demonstrate the convenience of SERS and the nature of non-destructive analysis.

### 2.4. Spectral Statistics Characteristics

Raman spectral information is of vital importance for revealing subtle analyte details [116,117]. The related statistical analysis involves preprocessing Raman data to isolate diagnostic bands, constructing classification or predictive models, and subsequently deploying the models for analyte determination. Remarkably, data preprocessing is essential for eliminating spectral noise, artifacts, and irrelevant signals arising from environmental factors and instrumental imperfections [72,89,118]. The used techniques include spike removal, wavenumber calibration, intensity normalization, smoothing, background subtraction, normalization, and dimensionality reduction.

Chemometric analysis of Raman spectra facilitates the extraction of distinct information for sample classification and differentiation. According to the availability of prior knowledge, the models are categorized into unsupervised and supervised pattern recognition. In both, unsupervised pattern recognition, such as principal component analysis (PCA), uncovers underlying structures within the unlabeled data and is often paired with clustering algorithms for sample classification. Supervised pattern recognition constructs a mathematical model based on a dataset of known categories, enabling the classification of unknown samples. Popular discriminant methods consist of linear discriminant, partial least squares discrimination, support vector machines (SVMs), and artificial neural networks [119].

Additionally, predictive models ascertain the relationship of variables to forecast the parameters of new observations. The selection of an appropriate mathematical model is critical, and consequently, various evaluation metrics have been developed to refine model parameters and assess performance. For example, the determination coefficient (R^2^) and root mean square error (RMSE) are used for optimizing model accuracy and reliability.

## 3. Sensitizing Effect of Nanostructures in the SERS Assay

With the rapid development of nanotechnology, nanostructures have been endowed with outstanding magnetic, electrical, optical, mechanical, and catalytic properties [120,121,122,123], and consequently have attracted great attention as a SERS strategy for signal enhancement [124,125,126].

### 3.1. Classification and Preparation of Nanostructures in SERS

#### 3.1.1. Classification of Nanostructures for Enhancing SERS Signals

The performance of SERS substrates is dependent on their ability of Raman signal enhancement. Fortunately, nanostructures can meet the demand for the performance improvement of SERS analysis due to their unique optical properties. As shown in Table 1, the used nanostructures in the SERS strategy comprise the following categories [127,128,129].

As confirmed, the used nanostructures significantly enhance the intensity and sensitivity of SERS signals by providing abundant “hot spot” areas, increasing the adsorption of target molecules, and adjusting surface plasmon resonance behavior [146,147,148].

#### 3.1.2. Preparation of Nanostructure-Sensitized SERS Substrates

As a mode dependent on the interaction of a rough metal surface with the target molecule for the signal output, the sensitivity of SERS often relies on metal-based nanostructures. The preparation methods [21,75,149] of nanostructure-sensitized SERS substrates mainly include the following.

1. Sol–Gel Method [99,150,151]

The sol–gel method can be used to prepare metal nanoparticles or nanocomposites, allowing control over particle size, morphology, and distribution on the SERS sensing platform. By adjusting the metal precursors and reductants in the solution, nanoparticles of various shapes (such as spherical, rod-like, etc.) can be synthesized.

2. Chemical Reduction Method [131,152]

The chemical reduction method typically uses the reductant to convert metal salts into metal nanoparticles on the SERS platform. For instance, the reductants, such as citric acid, sodium borohydride, and hydrogen gas, are used to prepare gold, silver, or other metal nanoparticles. The size and morphology of these nanoparticles can be controlled by adjusting the concentration of reductants, reaction time, temperature, etc. [153].

3. Photolithography and Electron Beam Etching Methods [125,154]

These methods are commonly used to prepare nanostructures with a precise morphology and size, such as metal nanorods and nanocolumns. They can precisely control the morphology of the SERS substrate, thereby enhancing the assay performance.

4. Template Method [155,156]

Using polymer templates, silica templates, etc., metal nanoparticles with regular structures can be prepared for the SERS design. The template method can prepare the desired metal structures through solvent removal, chemical etching, and is often used to manufacture periodic nanostructures.

5. Thermal Evaporation Method [157,158]

The thermal evaporation method involves heating metal materials to evaporate them and depositing them on a cooled substrate to form metal nanoparticles. This method can be used to prepare gold and silver films, and, consequently, is commonly used for the preparation of SERS substrates.

6. Solution Chemistry Method [41,152,159]

The solution chemistry method includes reduction reactions in a metal salt solution and SERS surface modification. By changing the pH value, temperature, and reaction time, metal nanoparticles can be prepared with different shapes and sizes.

7. Electrochemical Deposition Method [99,160]

The electrochemical method reduces metal ions to form nanoparticles on the sensing platform through electrolysis and electrodeposition. This method with good controllability is suitable for the preparation of large-area SERS substrates.

8. Self-Assembly Method [130,161]

The self-assembly method utilizes the self-assembling properties of molecules, nanoparticles, or polymers to generate structures with ordered arrangements on the sensing platform. This method is often used to manufacture nanostructures with specific shapes and morphologies, suitable for enhancing the Raman effect.

Generally, the performance of SERS substrates depends on the selection, morphology, and surface structure. Metal nanoparticles, metal nanorods, metal nanoshells, two-dimensional materials, and carbon nanostructures are widely used in the design of SERS substrates. Various synthesis methods, such as chemical reduction, template methods, and photolithography, can control the morphology, shape, and distribution of nanostructures as needed, further enhancing the SERS effect.

### 3.2. Enhancement Behavior of Nanostructure-Sensitized SERS

Benefiting from the merits of nanostructures, the Raman signal can be enhanced by the following behavior, including electromagnetic enhancement, chemical enhancement, and “hot spot” formation. The properties of nanostructures and measurement models also influence the Raman effect.

#### 3.2.1. Electromagnetic Enhancement Mechanism

**Figure 3:**
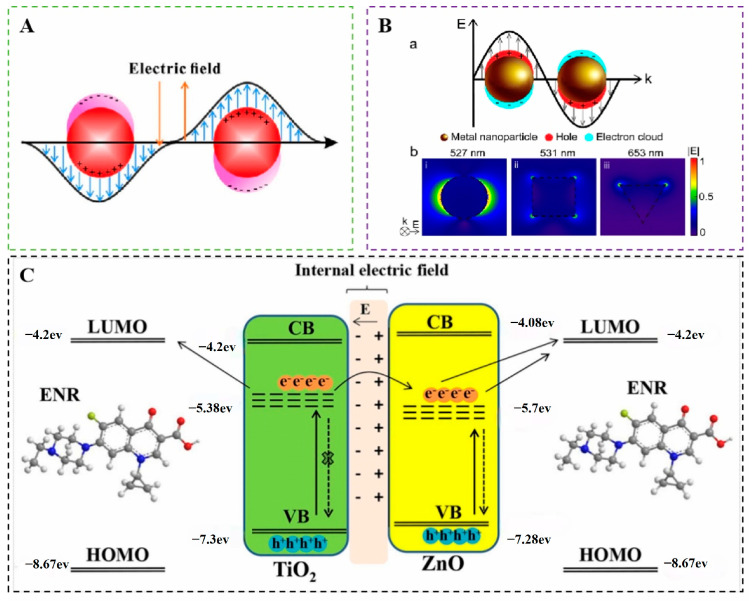
(**A**) Illustration of electromagnetic mechanism [1]. (**B**) Localized surface plasmon resonance on plasmonic nanoparticles. (**a**) Scheme of the electron clouds oscillating opposite from the direction of electric field close around the NPs with a size much smaller than the incident wavelength. (**b**) Finite difference time domain calculation of the on-resonance (wavelength labeled on the top of each figure) normalized electric field (|E|) distribution of (**i**) a nanosphere, (**ii**) a nanocube, and (**iii**) a nanotriangle [91]. (**C**) chemical mechanism for SERS. HOMO, highest occupied molecular orbital; LUMO, lowest unoccupied molecular orbital; CB, conduction band; VB, valence band [162].

The EM mechanism is an important way to enhance signals. When the incident light shines on the metal substrate surface, the free electrons on the metal surface oscillate collectively. When the generated electromagnetic wave frequency matches the incident light vibration frequency, a near-field electromagnetic wave propagates along the metal surface, resulting in SPR. A significantly enhanced electromagnetic field is formed on the metal surface (Figure 3A). If this electromagnetic field is confined to a small area of the metal surface, it is called LSPR. LSPR can significantly amplify the Raman signal of molecules in the vicinity. For the metallic nanostructure, the quasi-free electron clouds are pulled back and forth harmonically with the incident EM field against the Coulombic force between the electron and the nuclei (Figure 3(Ba)), leading to an enhancement of the localized EM field. The plasmonic nanostructures with sharp tips, narrow gaps, and aggregates (Figure 3(Bb)) generally provide more enhanced Raman scattering and their LSPR wavelengths can be tuned by factors, e.g., nanoparticle composition, size, alloy proportion, etc.

#### 3.2.2. Chemical Enhancement Mechanism

As one enhancement behavior of nanostructure-sensitized SERS, the CM mechanism involves CT between the sensing substrate and adsorbed molecules. The enhancement effect typically ranges from 10^1^ to 10^2^. This includes non-resonant enhancement, resonant enhancement, and photo-induced CT. As shown in Figure 3C, electrons are excited from the valence band of TiO_2_ and ZnO to their surface state energy levels (Ess), and then transfer to the lowest unoccupied molecular orbital (LUMO) of the Enrofloxacin (ENR) molecule for SERS signal enhancement. The Ess of ZnO is lower than that of TiO_2_, facilitating the injection of photoexcited electrons from TiO_2_ into ZnO and the subsequent transfer to ENR’s LUMO, forming a “C”-shaped CT pathway. Concurrently, photogenerated holes in ZnO’s valence band can migrate to TiO_2_. This process effectively inhibits an electron-hole recombination in TiO_2_, providing additional CT to adsorbed molecules and amplifying the SERS signal. Collectively, due to a strong interfacial interaction and high carrier concentration, efficient carrier separation from the heterostructure also contributes to the enhanced SERS signal.

#### 3.2.3. “Hot Spot” Formation

**Figure 4:**
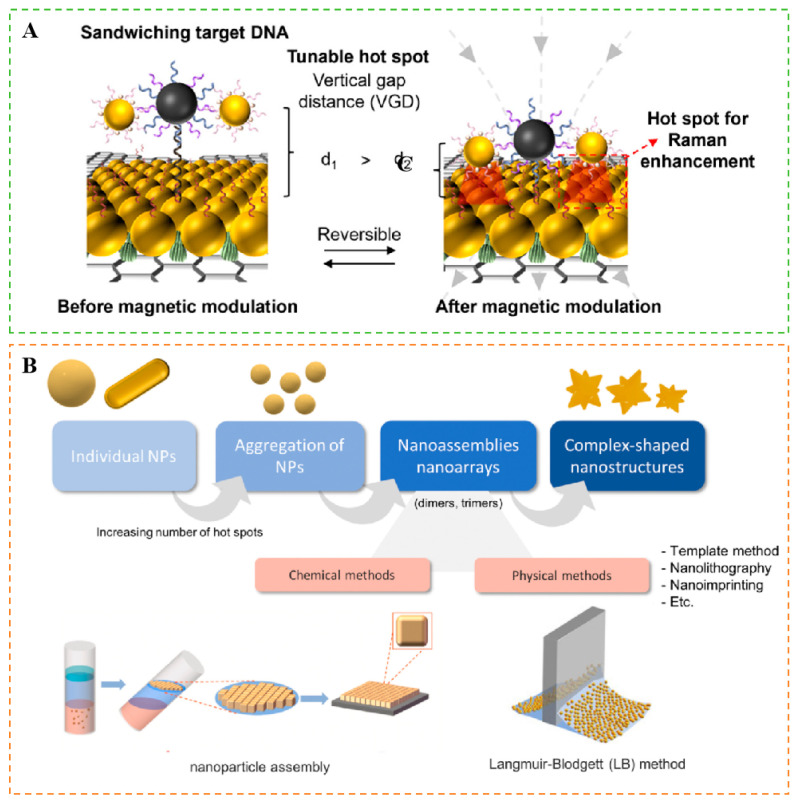
(**A**) Schematic diagram of the magnetic regulation of nanoparticle gaps [164]. (**B**) Scheme of the strategies to increase the density of hot spots and, thus, the enhancement of the SERS signal, namely the aggregation of individual nanoparticles; formation of dimers, trimers, nanoassemblies, and nanoarrays; and, finally, tailoring their shape into anisotropic and complex-shaped nanostructures [166].

In specific regions of metal nanostructures, such as the tips, edges, or gaps, the intensity of the electromagnetic field is particularly high [163,164]. These areas are referred to as “hot spots”. When molecules are located in these “hot spots”, Raman signals are greatly enhanced, which has a significant effect on improving SERS. By developing plasmonic nanoparticles with specific structures, such as nanostars and nanowires, local electric field confinement is used to amplify signals and large three-dimensional (3D) structures, such as ordered nanoaggregates or arrays, are constructed to generate a large number of nanogaps. These couplings produce SERS hot spots with greater SERS enhancement than zero- and one-dimensional nanostructures. Magnetic nanoparticles can be controlled by an external magnetic field to achieve directional motion and aggregation, providing a more flexible way of working [165]. Magnetic nanoparticles are combined with plasmonic nanoparticles to reconstitute SERS hot spots by magnetic aggregation or a magnetic adjustment of the gap. The magnetic adjustment of a nanoparticle gap is also a method to regulate the hot spot. Yin et al. used the external magnetic field to control the coupling distance of nanoparticles to achieve hot spot construction. The magnetic response platform and sensing platform were prepared in the experiment. By applying a magnetic field in a certain direction, the magnetic response platform was brought closer to the sensing platform, thus shortening the coupling distance and forming new hot spots between the plasma nanoparticles of the two platforms (Figure 4A). It overcomes the disadvantage that the expansion of the coupling distance between the two structures of the common sandwich method weakens the magnetic field intensity and reduces the signal. Under the effect of magnetic regulation, the SERS signal increased in the range of 1.4–1.8 times [164].

#### 3.2.4. Effect of Nanostructure Morphology and Composition on Signal Enhancement

**Figure 5:**
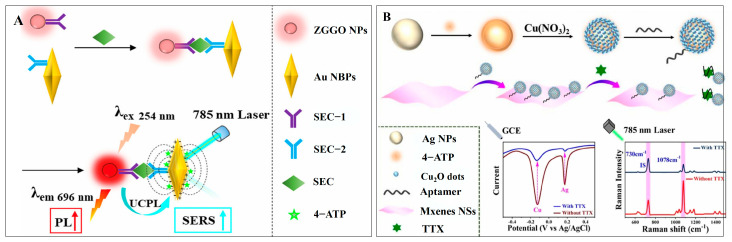
(**A**) Schematic illustration of the construction of the “Add on” dual-modal optical immunoassay [170]. (**B**) Schematic illustration of electroactive and SERS-Active Ag@Cu_2_O NP-engineered electrochemical/SERS dual-mode aptasensors [171].

The size and shape of nanostructures have a significant impact on their SERS activity [167]. Nanostructures of an appropriate size and shape can optimize the enhancement of the electromagnetic field [168], thereby increasing the intensity of the Raman signal. The design of nanostructures, such as nanostars, nanorods, and nanoshells, can increase the number and intensity of “hot spots”, thereby enhancing the SERS signal. Figure 4B depicts different strategies for generating hot spots between nanoparticles, from the aggregation of chemically synthesized nanoparticles (from spheroids to anisotropic nanostructures) into nanoclusters or nanoaggregates, to chemical methods by nanoparticle assembly and the use of different physical methods to form dimers, trimers, and ultimately arrange these nanostructures in a more controlled pattern. Meanwhile, the chemical functionalization of nanostructures can enhance their interaction with target molecules, improving the sensitivity and selectivity of the Raman signal [169]. By modifying specific functional groups or biomolecules (such as antibodies and aptamers) on the nanostructure surface, the recognition of analytes and signal enhancement can be achieved. Zhao et al. [170] used the SEC antibody/antigen as a linker arm to design Au NBP-ZGGO NP nanocomposites. In the presence of SEC, the functionalized ZGGO NP of the SEC antibody (SEC-1) and the modified Au NBP of the SEC paired antibody (SEC-2) are assembled into a double-antibody sandwich structure (Figure 5A). The enhanced fluorescence signal is obtained due to the plasmon-enhanced fluorescence mechanism. Au NBP with a sharp tip not only creates a “hot spot” for SERS performance, but also shows amplified SERS activity after its assembly with ZGGO NPs. ZGGO NPs were found to emit upconversion fluorescence, which further excites the plasmon resonance of Au NBPs, which mainly contributes to SERS enhancement. SEC antibody/antigen-engineered Au NBP-ZGGO NP nanocomposites have enhanced fluorescence signals and provide strong SERS activity, showing potential applications for “additional” dual-mode SEC detection.

#### 3.2.5. Multi-Modal Analysis of Signal Enhancement

With the help of nanostructures, SERS can be integrated with other detection modes (such as fluorescence, electrochemistry) to perform a multi-modal analysis, which can enhance Raman signals by leveraging the multiple functionalities of nanostructures. Yao et al. [144] found that Ag@Cu_2_O NPs modified by 4-ATP due to EM enhancement and CT mechanisms at the Ag and Cu_2_O junctions exhibited amplified SERS activity. They assembled aptamer-modified Ag@Cu_2_O NPs on the surface of MXene NSs. These components exhibit unique electrooxidation peaks and SERS signals. Adapted sensors for electroactive and SERS active Ag@Cu_2_O NPs engineering were then developed to achieve accurate dual-mode TTX detection (Figure 5B). Compared with the most reported single-signal detection method, the multi-channel biosensing platform with an independent detection signal improves the detection accuracy and reliability, and has great practical application potential in quantitative analysis applications. To be more specific, the electrochemical sensing interface can adsorb or desorb charged substances, allowing for maximum sensing area coverage or desorption of the target. The reproducibility of the SERS substrate is greatly improved.

In summary, nanostructures can enhance the Raman signal through multiple mechanisms, including the SPR effect, electromagnetic enhancement, chemical enhancement, formation of “hot spots”, size and shape effects, surface functionalization, and the combination with other detection modes, thereby achieving the high sensitivity and high selectivity of the SERS platform.

## 4. Nanostructure-Sensitized SERS for Assessing Harmful Substances in Food

Food safety incidents not only pose a threat to consumers’ health, but also severely hinder the progress of the food supply chain. It is widely acknowledged that the development of rapid and reliable technologies ensures the timely identification and control of food hazards. Among the developed patterns, SERS holds potential advantages for non-destructive and rapid detection in food safety evaluations. The physicochemical properties of SERS substrates play important roles in sensing performance [163]. However, traditional SERS substrates based on single noble metals suffer from poor sensing stability, limited specificity, and therefore are not suitable for sensitive detection in complex food scenarios. With the emergence of various functional nanostructures, the development of nanohybrid-sensitized platforms represents a promising frontier for SERS technology in high-sensitivity, interference-free, multiplexing, and reliable food monitoring. The advantages of nanostructure-sensitized SERS for the detection of hazardous substances in food are shown in Table 2.

With the merits, nanostructure-sensitized SERS offers a highly effective tool in the field of food safety testing. However, its complex preparation process, high costs, poor material stability, insufficient signal uniformity, and environmental sensitivity limit its large-scale application. Therefore, while the nanostructured SERS substrate exhibits a strong detection performance, further optimization is needed in preparation processes, cost control, and stability enhancement to fully realize its application potential.

### 4.1. Nanostructure-Sensitized SERS to Assess Microbial Contamination in Food

Microbiological contamination remains an urgent issue in the global food safety domain [172,173]. Such contamination encompasses bacteria, viruses, fungi, and parasites, potentially originating from cross-contamination during agricultural production, processing, or transportation [174]. Common foodborne pathogens include *Salmonella*, *Listeria*, *E. coli*, and *Staphylococcus aureus* (*S. Aureus*) [175,176]. Outbreaks of foodborne diseases not only impact public health but also result in significant economic losses. Traditional microbial assay methods, such as culture-based testing and the polymerase chain reaction (PCR) [177,178], are highly accurate but often time-consuming, requiring complex preparation methods. To enhance assay efficiency, the researchers have developed rapid methods based on technologies like SERS, ELISA, and electrochemical sensors. In particular, SERS technology has shown immense potential for microbial detection due to its high sensitivity and specificity. Employing metal nanostructures as sensing platforms, SERS is capable of detecting microbial markers at the single-molecule level. Notably, SERS technology can be applied in non-aqueous environments, which is particularly important for monitoring microbial presence in low-moisture foods.

#### 4.1.1. Sensing Foodborne Pathogens

Foodborne pathogens are bacteria that can use food as a carrier and produce harmful effects on the body after consumption [179,180]. Foodborne pathogens are considered as the main cause of food poisoning and infectious disease epidemics. Therefore, the effective detection of foodborne pathogens is indispensable for safeguarding public health safety. Pan et al. [26] developed a gold nanorod-based SERS substrate for tracking *E. coli* in low-moisture foods, in which the sensing platform was prepared by physical stamping, magnetron sputtering, and electrochemical deposition. *E. coli* was cultured to a concentration of 10^3^ CFU/mL and then mixed with ground black pepper and egg powder to prepare samples with contamination levels of 10^2^ CFU/g and 10^3^ CFU/g, respectively. A total of 0.5 mL of SERS nanoprobes (4-ATP-functionalized gold nanorods) was added to each sample and incubated at 4 °C for 30 min to bind the nanoprobes to *E. coli* antibodies, forming SERS nanoprobes that could specifically recognize bacterial cell surface antigens. The enhancement effect is orders of magnitude stronger than the unamplified signal (SERS enhancement in bacterial cells is typically 10^5^–10^6^). Moreover, magnetic separation techniques have also been used to capture *E. coli* and Staphylococcus aureus in samples with an enhancement factor (*EF*) of up to 10^6^ on the SERS platform and have been successfully applied to milk and beef samples [181,182].

The enhancement of the SERS signal mainly depends on the “hot spot” effect generated by the surface of the nanomaterial and the charge transfer mechanism. Precious metal nanoparticles, such as Au and Ag, are widely used in SERS substrates due to their surface plasmon resonance effect, which can significantly enhance the Raman signal. In addition, 2D materials, such as GO, further enhance the signal enhancement effect through the synergy with metal nanostructures. In the above studies, the SERS signal enhancement factor generally reached 10^5^–10^6^, indicating that these materials have extremely high sensitivity in the detection of foodborne pathogens. The SERS-based method for the detection of foodborne pathogens achieves a highly sensitive detection of pathogens at low concentrations by combining functionalized nanomaterials and specific recognition probes.

#### 4.1.2. Sensing Fungi, Molds, and Their Toxins

**Figure 6:**
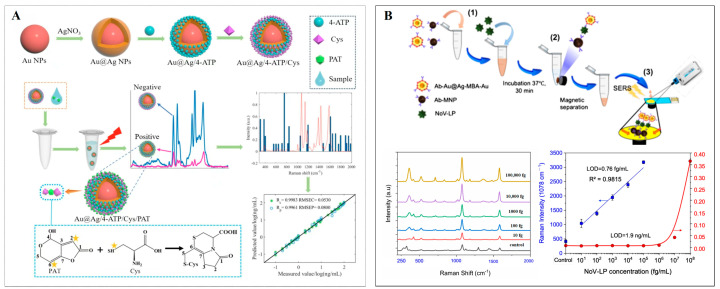
(**A**) Schematic diagram of SERS for patulin based on the NAR-SERS substrate (Au@Ag/4-ATP/Cys) [132]. (**B**) Detection of norovirus-like particles in PBS using the Palmtop Raman spectrometer [34].

In recent years, SERS technology has shown great potential for the detection of mycotoxins, such as patulin (PAT) and aflatoxin B1(AFB1). Ma et al. [132] used a cysteine (Cys)-mediated nucleophilic addition reaction combined with chemometric algorithms to achieve the rapid and sensitive detection of PAT in apples. PAT standard powder was dissolved in acetonitrile and prepared as a 100 μg/mL standard solution. The citrus samples without PAT contamination were crushed and filtered to obtain a clear solution. PAT standard solutions of different concentrations were added to the clarification solution to prepare a sample solution containing PAT. Ethyl acetate was added to the CAT clarifier, shaken and sonicated, centrifuged, and the supernatant was dissolved in acetonitrile as the PAT solution to be tested. The solution containing PAT was mixed with a NAR-SERS substrate (Au@Ag/4-ATP/Cys) and the SERS spectra were acquired by a Raman spectrometer (638 nm laser). The surface plasmon resonance effect of Au@Ag NPs generates a strong electromagnetic field under laser irradiation, which enhances the Raman scattering signal of nearby molecules. Combined with chemometric algorithms, hidden spectral information can be extracted from the spectral data more efficiently, and the prediction performance of detection can be improved (Figure 6A). In citrus samples, the recovery of the SERS method was in the range of 96.58% to 101.74%, which was consistent with the results of the HPLC method (*p* = 0.283 > 0.05), indicating the good accuracy and applicability of the method.

Multimodal aptamer sensors are also used to detect mycotoxins [183]. Xie et al. [184] developed a three-mode (colorimetric, photothermal, and surface-enhanced Raman scattering) lateral flow immunoassay (LFIA) based on a Hollow Porous Gold Nanoflower (HPGN) for the detection of AFB1. Prepare 0.1–10 ng/mL AFB1 standard solution using PBS buffer. Uncontaminated corn and wheat flour were mixed with PBS, centrifuged, and the supernatant was diluted 50-fold for testing. The reliability of the method was assessed by adding 0.2, 0.5, and 1 ng/mL of AFB1 to the samples. SERS detection of AFB1 was achieved by modifying 4-MBA on the surface of the HPGN. The porous structure and large specific surface area of the HPGN significantly enhanced the Raman signal. The detection limit of the SERS mode was as low as 0.05 ng/mL and was successfully applied to the detection of AFB1 in maize and wheat samples.

In all these studies, SERS signal enhancement factors are generally high, such as Au@Ag enhancement effects of nanoparticles and HPGNs, resulting in a detection sensitivity of 0.1 pg/mL or even lower. The detection method based on SERS technology achieves the high sensitivity detection of trace toxins by combining functionalized nanomaterials and chemometric algorithms.

#### 4.1.3. Sensing of Viruses

The researchers constructed core–shell satellite nanoparticles as signal amplification nanotags to achieve the highly sensitive detection of viruses [185]. Y Park et al. [34] performed a dilution experiment using stool samples from patients infected with the norovirus (NOV). The sample solution was added dropwise to the gold electrode and detected using a handheld Raman spectrometer. The system ranged from 10 fg/mL to 100 pg/mL for norovirus-like particles (NoV-LPs) with a limit of detection (LOD) of 0.76 fg/mL. The Au@Ag-MBA-AuNPs nanotags had an enhancement factor (*EF*) of up to 10^8^, indicating that they had extremely high SERS activity (Figure 6B). Moreover, a low-temperature stable SERS substrate (Ag@ICNPs) was developed for the highly sensitive detection of monkeypox virus [186]. This study successfully obtained stable SERS signals of biomolecules at −20 °C low-temperature conditions, which was particularly important for the virus assay on cold chain food packaging. The system has a linear range of 6.25 × 10^3^ to 10^5^ copies/mL and a LOD of 6.25 × 10^3^ copies/mL.

In the above studies, the *EF* was as high as 10^8^, which significantly improved the detection sensitivity. The virus nucleic acid detection method based on SERS technology achieves the high sensitivity detection of trace viral nucleic acids by combining functionalized nanomaterials and signal amplification strategies. In the future, with the further development of nanomaterial design and low-temperature detection technology, SERS technology will be more widely used in the field of virus detection, especially in the detection of cold chain food and clinical samples.

### 4.2. Nanostructure-Sensitized SERS Assessing Chemical Contamination in Food

The effective analysis of chemical contamination is an essential component of food safety, involving the assay of pesticides, veterinary drugs, and their metabolites in agricultural and livestock products [187]. Technologies [188,189,190] such as high-performance liquid chromatography (HPLC), gas chromatography (GC), liquid chromatography-mass spectrometry (LC-MS), and gas chromatography-mass spectrometry (GC-MS) have been widely applied in monitoring chemical contamination. Emerging detection technologies like SERS have garnered attention for their high sensitivity and rapid response capabilities. However, enhancing the sensitivity and selectivity of the Raman pattern for certain low-concentration or structurally similar compounds remains a challenge. With the advancement of chemometrics and machine learning technologies, the data analysis capabilities of Raman spectroscopy have been strengthened, improving the assay’s accuracy [191,192]. More comprehensive Raman spectroscopy databases and standard libraries could support the identification and quantification of more compounds. The efficient sample pretreatment and analysis methods offered useful tools for the simultaneous detection of multiple agrochemical and veterinary drug residues at the ppt (part-per-trillion) level [163,193].

#### 4.2.1. Sensing of Pesticide Residues

**Figure 7:**
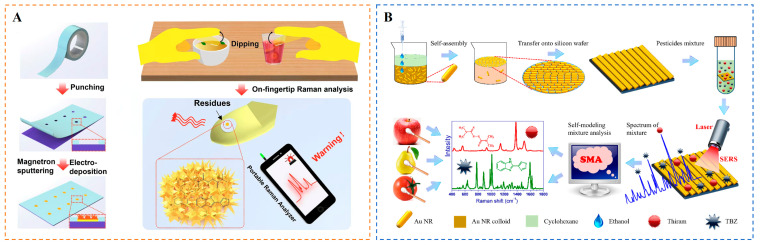
(**A**) Tape-based SERS sensors for the on-hand detection of food contaminants [194]. (**B**) Schematic of interfacial self-assembly of Au NR array for multiple pesticides on fruit surface based on SERS and self-modeling mixture analysis [130].

For the detection of pesticide residues in food, the detection substances are mostly thiabendazole and thiazole. Both He et al. [194] and Hu et al. [130] utilized special gold nanomaterials as SERS substrates to enhance the Raman signal, He et al. employed Au nanodendrites with sharp edges and tips, while Hu et al. used high-density gold nanorod (Au NR) arrays. They all enriched the target pesticide residues by specific methods (capillary action micropore strips to capture droplets, Hu et al.’s modified swab extraction method) to improve the detection sensitivity. In addition, both achieved good enhancement factors, with the *EF* of Au NR arrays of about 10^8^ in He et al.’s study and 1.64 × 10^6^ in Hu et al.’s study, and both successfully achieved the effective detection of pesticide residues in actual food samples (Figure 7). In addition, a three-dimensional “hot spot” structure SERS substrate was used for the quantitative detection of pesticide residues (tetramethylthiuram disulfide (T1), methylene blue (MB), methyl viologen dichloride (MV), and malachite green (MG)) on food surfaces [84]. The SERS substrate was designed by assembling gold cores on polystyrene (PS) microspheres, modifying the surface with a Raman internal standard (1,4-benzenedithiol(1,4-BDT)), and subsequently growing a gold shell on this internal standard. Three-dimensional hot spot regions were constructed, primarily located between the hydrophobic gold film and the composite particles, as well as between the particles and within the core–shell structure. The enhancement of the SERS signal is mainly due to the electromagnetic effect, and local surface plasmon resonance occurs when the frequency of the light matches the oscillation frequency of the electrons. The hydrophobic interface facilitated the enrichment and capture of analytes. The PS@Au@1,4-BDT@Au solution was mixed with the sample to be tested and added dropwise to the hydrophobic gold film. After the samples were dried on a hydrophobic gold film, they were detected using a portable Raman spectrometer. This mode demonstrated high sensitivity and stability in tracing pesticide residues on the surfaces of cucumbers, apples, and fish, beneficial for a low-cost and effective SERS platform for various pesticide residues. By comparison with conventional Raman spectroscopy, the *EF* of the PS@Au@1,4-BDT@Au substrate reaches about 10^8^.

In the above studies, the EFs of the SERS signal were generally high, such as 10^8^ for Au nanodendrites, which significantly improved the detection sensitivity. The pesticide residue detection method based on SERS technology performs the highly sensitive detection of trace pesticides by combining functionalized nanomaterials and optimizing the substrate structure.

#### 4.2.2. Sensing Veterinary Drug Residues

Several studies have used SERS technology in combination with specific nanocomposites to perform the highly sensitive detection of veterinary drug residues (Chloramphenicol (CAP)) in food samples. These uniquely structured nanomaterials (e.g., spiny Au@Ag nanoparticles [42], Fe₃O₄-WO₃-X@AuNPs [195], MDAu@Ag [196], etc.) and composite materials (e.g., Bi₂WO₆ composite films [193]) significantly amplify SERS signals through EM and CM mechanisms. At the same time, these studies have further optimized the detection performance through specific preparation methods (such as the chemical reduction method, self-assembly method, etc.) and structural design (such as the construction of hot spots, the introduction of magnetic cores, etc.). In addition, these methods have been validated in actual samples (e.g., milk, cabbage, meat, etc.) and have shown good accuracy and sensitivity, and the detection limits are well below the maximum residue limits specified in the relevant standards, providing an effective detection method for food safety monitoring.

#### 4.2.3. Sensing of Heavy Metals

Heavy metal ions easily enter food and drinking water through natural environmental pollution, industrial emissions, and chemical fertilizers [49,197,198]. Owing to the high toxicity, easy enrichment by bioaccumulation, the non-degradation of heavy metals, it is necessary to develop effective strategies for heavy metal monitoring [199,200,201]. SERS technology combined with specific nanocomposites has shown a highly sensitive detection of different metal ions (Hg^2^⁺, Mn^2^⁺, Cd^2^⁺) [133,202,203]. Dong et al. [152] proposed a novel magnetic SERS tag (Fe@RAu) for ultrasensitive immunochromatographic detection (ICA) to monitor cadmium ions (Cd^2+^) in complex samples. The combination of a large Fe_3_O_4_ core and a coarse gold shell provided a robust magnetic response and high SERS activity, enabling the fast, low-cost sensing of heavy metal contamination in aqueous environments. Milk and pork extracts were used as actual samples in the experiment. The sampling process was as follows: Milk samples were taken directly from pure milk without any pretreatment. A total of 10 g of pork was treated, weighed and dispersed in 10 mL of PBS solution (10 mM, pH 7.4), sonicated for 5 min, then filtered through 2 μm filter paper, and the supernatant was collected for subsequent detection. Milk and pork extracts were supplemented with different concentrations of Cd^2+^ (3 ng/mL, 0.3 ng/mL, and 0.03 ng/mL) to verify the practical application of the assay method. The detection limit of Cd^2+^ was 1.88 pg/mL, which was well below the maximum allowable level of Cd^2+^ in actual samples set by the Codex Alimentarius Commission. The Fe@RAu magnetic SERS tag has an *EF* of 5.25 × 10^6^.

#### 4.2.4. Sensing Food Additives and Illicit Adulterants

**Figure 8:**
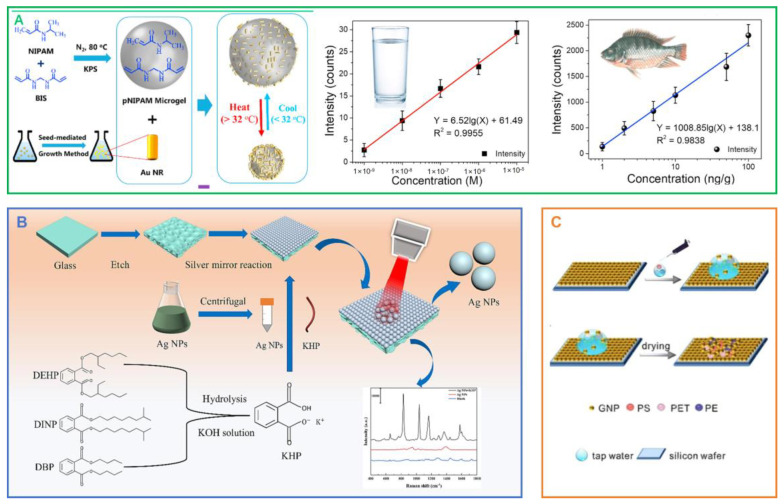
(**A**) Fabrication and characteristic procedures of pNIPAM, Au NRs, and pNIPAM@Au NRs; SERS intensity of MG in water and in fish tissue extract at 1618 cm^−1^ as a function of the logarithmic MG concentrations [208]. (**B**) Schematic diagram of SERS sensing of plasticizer in oil [209]. (**C**) Illustration of liquid interfacial coassembly plasmonic array (LICA) of plasmonic GNPs and nanoplastics [17].

Food additives are a type of natural or chemical synthetic substance used to improve the food flavor, texture, and storability. Normal use within the permitted range does not cause harm to humans, but exceeding usage limit can be toxic and carcinogenic to humans [204,205]. Common food additives and illegal additives are rhodamine 6G (R6G), crystal violet (CV), MG and phthalates (PAEs), and 2,6-di-tert-butyl-p-cresol (BHT) [206,207]. Composites of polymeric polyN-isopropylacrylamide (pNIPAM) and Au NRs have been studied for the detection of MG in water and fish tissues (Figure 8A). After tilapia fillets were homogenized, different concentrations of MG standard solutions were added for spike experiments. Ammonia was used to prevent MG degradation, acetonitrile and anhydrous magnesium sulfate were added for extraction, and then the metabolites of MG were oxidized to MG with the oxidant 2,3-dichloro-5,6-dicano-1,4-benzoquinone (DDQ), and finally SERS detection was performed by 0.22 μm filter membrane filtration. The limit of detection for MG in the aqueous solution was 3.95 × 10^−10^ M and 0.73 ng/g (1.58 × 10^−9^ M) in the fillet tissue [208]. Wang et al. [209] performed the highly sensitive detection of PAE by designing a specific combination of two-dimensional (2D) silver plates and nano-silver sols (Figure 8B). Edible oil samples (e.g., camellia oil) were dissolved in ethanol, and a potassium hydroxide solution was added and reacted in a 75 °C water bath for 30 min to hydrolyze the PAE into water-soluble potassium hydrogen phthalate (PHP). The hydrolyzed solution was diluted 10^4^ times, and then the nano-silver sol was mixed with the PHP solution, and then dropwise added to the surface of the 2D silver sheet, which was naturally dried and then detected by SERS. The minimum detection limit for PHP is 10^−9^ mol/L. This silver plate provided a homogeneous metal surface that amplified the Raman signal of the sample molecules. The sol of silver nanoparticles was used as a synergistic agent with 2D silver plates to further enhance Raman scattering. The addition of silver nanoparticles increases the number of hot spots in the system, thereby increasing the intensity of the Raman signal.

The above results show that the Raman signal can be significantly enhanced and the detection limit can be reduced by using the nanogap effect by rationally designing the SERS substrate material and optimizing the structure, so as to achieve the high-sensitivity detection of chemical contaminants in food. Spike-like structures, magnetic materials, and nanogap designs can significantly enhance the SERS signal and reduce the detection limit. The successful application of these platforms not only proves the potential of SERS technology in actual sample testing, but also provides a new direction for the development of future food safety monitoring.

### 4.3. Nanostructure-Sensitized SERS to Assess Physical Contamination in Food

Physical contamination in food refers to the pollution of food by non-biological and non-chemical substances, which may affect the safety and quality of food and even pose a threat to human health. Physical pollution comes from a variety of sources, including a variety of factors in the process of agricultural production, processing, transportation, storage, and marketing. Some of the common species include glass shards, stones, bugs, contamination of packaging materials, etc. [210,211,212].

Microplastic, as a kind of plastic particle smaller than 5 mm but larger than 0.1 µm, is considered as a threat to foods and beverages. the identification of microplastic particles from complex matrices can be challenging due to their various sizes, shapes, and polymer types. Yu et al. [17] exploited a liquid–liquid interface strategy to assemble micro-/nanoplastics and gold nanoparticles (GNPs) into dense and uniform plasma arrays (Figure 8C), thereby enabling the rapid and sensitive detection of trace nanoplastics. Tap water and lake water were collected as environmental water samples, and polystyrene (PS) nanoplastic solutions with different concentrations were prepared. Buy edible oils (such as soybean oil and edible blended oil) and add different concentrations of PS nanoplastic solutions. In environmental water samples and edible oils, the detection limit of nanoplastics is 1 μg/mL. Moreover, ethyl acetate was selected as the organic phase to participate in the self-assembly process due to its polarity being close to that of water, which helped to accelerate the self-assembly process and produce the strongest SERS signals. The presence of micro-/nanoplastics effectively promoted the self-assembly process of GNPs at the oil–water interface, increasing the number of molecules in hot spots for SERS signal amplification. The aromatic rings in the micro-/nanoplastics interacted strongly with the gold nano-surface through π-metal interactions, promoting the adsorption of more micro-/nanoplastics at the liquid–liquid interface. The PCA algorithm was utilized to process the SERS data to distinguish and identify various micro-/nanoplastics components in aquatic environments as well as in edible oils. SERS technology has shown a broad application prospect in the detection of physical contamination of food. In the future, it is expected to be applied to the detection of other physical contaminants.

In conclusion, nanostructure-sensitized SERS platforms have shown remarkable properties in food hazard analysis. This article summarizes its latest applications for the detection of food hazards and classifies them by common contaminants (Table 3).

## 5. Conclusions

In this review, we discuss the important role of nanostructures in enhancing Raman signals, in particular the application of nanostructure-sensitized SERS in food safety evaluations. By analyzing the latest research findings, this review summarizes the critical role of nanostructures in SERS technology and how they can help improve efficiency and accuracy. In addition, nanostructures facilitate the application of SERS technology in combination with other detection technologies, providing an efficient and low-cost solution for food safety testing. The application of nanostructures in SERS technology not only improves the efficiency and accuracy of food safety assays, but also provides new ideas and directions for the development of food safety evaluation technology in the future. With the continuous progress of nanostructure technology and the continuous improvement of SERS technology, its application prospect in food safety testing will be broader.

## Figures and Tables

**Table 1:** Commonly used nanostructures in the SERS platform.

Classification	Examples	Specific Materials	Characteristics	References
Metal Nanomaterials	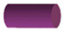	Au, Ag, metal alloy	Widely used in SERS substrates due to their excellent LSPR property.	[7,130]
Core–Shell Structure	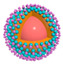	Au@Ag, Ag@Au	Optimizing SERS signals by adjusting the properties of the outer metal layer.	[13,131,132]
Porous Materials	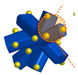	Porous carbon (PC), porous silicon (PS), MOFs	High specific surface area and good molecular sieving effects, effectively adsorbing target molecules and enhancing SERS signals.	[39,133,134]
Semiconductor Nanostructures	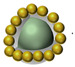	Titanium dioxide (TiO_2_), zinc oxide (ZnO)	Weak SERS activity on their own, but can enhance signal strength when combined with metal nanoparticles.	[13,135,136]
Carbon-Based Nanostructures	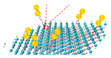	Graphene and its derivatives (e.g., reduced graphene oxide)	Excellent conductivity and large specific surface area, effectively enhancing SERS signals.	[137,138]
Polymer-Based Nanostructures	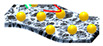	Functionalized polymers (e.g., PMMA, PDMS)	Combining with metal nanoparticles to form a composition for enhancing SERS signals.	[139,140]
Biomass-Based Nanostructures	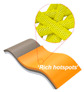	Natural polymers (e.g., chitosan, gelatin, cellulose nanofibers)	Biocompatibility and tunability allow them to combine with metal nanoparticles for enhancing signals.	[141,142,143]
Composite Nanostructures	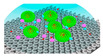	Combination of different nanostructures (e.g., metals with semiconductors, metals with porous materials)	Achieving synergistic effects to further enhance SERS signals.	[13,133,134,144,145]

**Table 2:** The advantages of nanostructure-sensitized SERS for the detection of hazardous substances in food [147,148,163].

Advantage	Description
High Sensitivity Detection	SERS technology uses the surface plasmon resonance of metal nanoparticles to significantly enhance the Raman signals of adsorbed molecules, enabling the highly sensitive detection of trace hazardous substances in food.
Rapid Response	SERS technology can provide rapid assay results, which is crucial for an immediate response and the management of food safety incidents.
No Need for Labeling and Pretreatment	SERS detection does not require complex sample pretreatment or labeling; it can directly test food samples, simplifying the operational process.
High Selectivity	SERS technology exhibits high selectivity, enabling the rapid quantitative or qualitative detection of hazardous substances in complex food matrices.
Multiplex Detection Capability	SERS technology is capable of detecting multiple hazardous substances simultaneously, enhancing the efficiency and scope of detection.
Real-Time Monitoring Capability	Combined with portable devices, SERS technology can perform the real-time monitoring of hazardous substances during food processing and storage.
Data Traceability	The Raman spectra provided by SERS have unique fingerprint characteristics, aiding in tracing contamination sources and food safety traceability.
Strong Environmental Adaptability	Nanostructures can be used under various environmental conditions, enhancing the application potential of SERS technology in diverse food testing scenarios.
Cost-Effectiveness	Although the initial investment may be high, SERS technology reduces the costs associated with repeated testing and erroneous results, making it cost-effective in the long run.
Biocompatibility	Selecting appropriate nanostructures ensures that SERS detection is safe for both the food and operators, avoiding secondary contamination.

**Table 3:** Application of the nanostructure-sensitized SERS platform in food safety detection methods.

Types of Food Contamination	Types of Nanostructures	SERS Substrate	*EF*	Analytes	LODs	Ref.
Microbial Contamination	Metal	Au NR		*E. coli*	10^2^ CFU/g	[26]
		AuNPs		*S. Aureus*	10 CFU/mL	[213]
		Au NPs	1.8 × 10^5^	*S. Aureus*	1.5 CFU/mL	[214]
		Ag-NDs	3.9 × 10^6^	*S. Aureus*	10^2^ CFU/mL	[215]
		HPGN		AFB1	0.05 ng/mL	[184]
		Ag@ICNPs	10^8^	Monkeypox Virus	6.25 × 10^3^ copies/mL	[186]
		Au NPs-DNA (H2)-DTNB Ag NRs-DNA (H1)		*S. Aureus*	5 CFU/mL	[216]
		Au-Ag nanodimer		*S. Aureus*	96 CFU/mL	[217]
	Core–Shell	Ag@AuNPs		HCV	0.47 fM	[185]
		Au@Ag/4-ATP/Cys		PAT	0.27 ng/mL	[132]
		Au@Ag-MBA-AuNPs	10^8^	NOV	0.76 fg/mL	[34]
		AuNSs@PB@Ag-Apt		*S. Aureus*	1 CFU/mL	[218]
		Au@AgPt		*S. Aureus*	6 CFU/mL	[219]
		Au@NTP@SiO₂-ssDNA	9.6 × 10^5^	*S. Aureus*	2 CFU/mL	[220]
	Porous Materials	Ag-pSi		*E. coli*	3 CFU/mL	[221]
		Ag-pSi-DTNB		*S. Aureus*	2 CFU/mL	[222]
	Biomass-Based	AgNPs@4-MB@MPNs	10^6^	*E. coli* O157:H7, *S. Aureus*	10^2^ CFU/mL	[182]
	Semiconductor	QD-SNPs DTNB-AuNPs		AFB1	0.100 pg/mL	[183]
		Au@Ag@MBA@SiO_2_		*S. Aureus*	2 CFU/mL	[223]
	Carbon-Based	Ti_3_C_2_Tx@Au NPs@4-MBN		*E. coli*	10 CFU/mL	[224]
	Composite	Fe_3_O_4_@SiO_2_-Au NPs		*E. coli* and *S. Aureus*	10 CFU/mL	[181]
		Fe_3_O_4_@Au		*S. Aureus*	25 CFU/mL	[225]
		Fe_3_O_4_@Au NFs		AFB1	0.40 pg/mL	[226]
		Fe_3_O_4_-Apt MNPs Van-Au NPs		*S. Aureus*	3.27 CFU/mL	[227]
		MnO₂@AuNPs		*S. Aureus*	1.561 CFU/mL	[228]
		ZnO/Ag–Au@Ag	4.67 × 10^5^	*S. Aureus*	10 CFU/mL	[229]
Chemical Contamination	Carbon-Based	GE-AuNPs/GO	1.92 × 10^9^	R6G	7.33 × 10^−11^ M	[230]
		AuNPs/NH_3_-GO	7.0 × 10^3^	R6G	10^−7^ M	[231]
		AgNFs-GO-AuNSts	2.59 × 10^7^	R6G	1.0 × 10^−13^ M	[232]
		MXene/AuNRs	4.5 × 10^6^	Thiram	1 × 10^−10^ M	[233]
		Al/f-C_3_N_4_/AgNWs	3.37 × 10^5^	MG	8.38 × 10^−12^ M	[234]
		Ag NPs@g-C_3_N_4_	1.4 × 10^8^	Hg^2+^	0.02005 ppm	[235]
	Composite	Spiky Au@Ag NPs-Bi₂WO₆ films	Spiky Au@Ag NPs-Bi₂WO₆ films (1.8 times), CuB₂O₄ (1.3 times), BiOI (1.1 times)	CAP, paraquat	8.46 ng/L (CAP), 75.71 ng (paraquat)	[42]
		^mAb^AuNp^DTNB^ nanoparticles		SM2	0.1 pg/mL	[193]
		Au-NiFe LDH/rGO		Hg^2+^	1 × 10^−8^ M	[236]
		Fe_3_O_4_-WO_3_-X@AuNPs	6.1 × 10^5^ (ABZ), 7.8 × 10^5^ (SPT)	ABZ, SPT	12.5 μg/L (ABZ), 11.6 μg/L (SPT)	[195]
		Fe_3_O_4_/GO/Ag	2.26 × 10^7^	MB	10^−9^ M	[237]
		Fe@RAu	5.25 × 10^6^	Cd^2+^	1.88 pg/mL	[152]
		Fe@Ag		Thiram	0.01 ppm	[238]
		BP/Ag/TNR	3.81 × 10^5^	R6G	1.0 × 10^−14^ M	[239]
		BP@Au-Ag	2.5 × 10^7^	Thiram	2.6 × 10^−6^ mg/mL	[240]
		pNIPAM@Au NRs		MG	3.95 × 10^−10^ M (aqueous solution), 0.73 ng/g (1.58 × 10^−9^ m, fillet tissue)	[207]
	Metal	Au DNs	4.2 × 10^6^	Triazolone	0.00011 μg/mL	[83]
		Au nanodendrite tape substrate	10^8^	Thiram, Thiabendazole	1 × 10^−5^ M	[194]
		Au NRs array	1.64 × 10^6^	TBZ, Thiram	TBZ (0.79 ng/cm^2^ on apple, 0.76 ng/cm^2^ on tomato and 0.80 ng/cm^2^ on pear), Thiram (0.041 ng/cm^2^ for apples, 0.029 ng/cm^2^ for tomatoes, and 0.047 ng/cm^2^ for pears)	[130]
		3D Au@Ag NPs	3.4 × 10^6^	Thiram	10^−6^ M	[241]
		MNA-MA-AgNPs		Mn^2+^	4.0 × 10⁻^6^ mol/L (220 μg/L)	[202]
		2D silver plate		PAE	10^−9^ mol/L	[208]
	Core–Shell	MDAu@Ag-based SERS-LFA		Kana, RAC, CLE, CAP	0.52 pg/mL (Kana), 2.5 pg/mL (RAC), 0.87 pg/mL (CLE), 6.2 pg/mL (CAP)	[196]
		Au@Ag-GU		Hg^2+^	0.33 ppb	[242]
	Porous Materials	Au@Ag/COF		Hg^2+^	5.0 × 10^−16^ M	[133]
	Semiconductor	AgNTs/TNA		R6G, CV, MG, Thiram	10^−10^ M(R6G), 10^−9^ M(CV), 10^−8^ M(MG), and 10^−7^ M(Thiram)	[206]
		ZNR/AgT	Z3/AgT (5.4 × 10⁷, comparison with a glass substrate containing only silver nanotriangles)	BHT	0.1 ng/mL	[209]
		TiO_2_ NTs/AgNPs-rGO	7.1 × 10^8^	MB	1.0 × 10^−14^ M	[110]
	Polymer-Based	PS@Au@1,4-BDT@Au	10^8^	MB, T1, MV, MG	10^−12^ M (MB), 2.5 × 10^−8^ M (T1), 10^−8^ M (MV), 10^−11^ M (MG)	[84]
Physical Contamination	Metal	GNPs		Nanoplastic components (e.g., PS, PE, PP)	1 μg/mL	[17]
		silver colloid	7 × 10^2^ (100 nm PS ball), 1.1 × 10^4^ (500 nm PS ball)	PS, PE, PP	40 μg/mL (100 nm plastic particles), 0.08 mg/mL (500 nm plastic particles)	[243]

## Data Availability

No new data were created or analyzed in this study. Data sharing is not applicable to this article.
